# Ubiquitin-specific peptidases in lymphoma: a path to novel therapeutics

**DOI:** 10.3389/fphar.2024.1356634

**Published:** 2024-11-27

**Authors:** Maryam Samareh Salavatipour, Shirin Tavakoli, Aram Halimi, Shima Tavoosi, Amir-Hossein Baghsheikhi, Abdolkarim Talebi-Taheri, Mehdi Niloufari, Zahra Salehi, Javad Verdi, Soheila Rahgozar, Alireza Mosavi-Jarrahi, Mohammad Ahmadvand

**Affiliations:** ^1^ Department of Applied Cell Sciences, School of Advanced Technologies in Medicine, Tehran University of Medical Sciences, Tehran, Iran; ^2^ Department of Epidemiology, School of Public Health and Safety, Shahid Beheshti University of Medical Sciences, Tehran, Iran; ^3^ Research Center for Social Determinants of Health, Research Institute for Endocrine Sciences, Shahid Beheshti University of Medical Sciences, Tehran, Iran; ^4^ Department of Cell and Molecular Biology and Microbiology, Faculty of Biological Science and Technology, University of Isfahan, Isfahan, Iran; ^5^ Department of Biology, Science and Research Branch, Islamic Azad University, Tehran, Iran; ^6^ Student Research Committee, School of Medicine, Shahid Beheshti University of Medical Sciences, Tehran, Iran; ^7^ Shahid Beheshti University of Medical Sciences, Tehran, Iran; ^8^ Hematology, Oncology and Stem Cell Transplantation Research Center, Research Institute for Oncology, Hematology and Cell Therapy, Tehran University of Medical Sciences, Tehran, Iran; ^9^ Cancer Research Centre, Shahid Beheshti University of Medical Sciences, Tehran Iran; ^10^ Cell Therapy and Hematopoietic Stem Cell Transplantation Research Center, Research Institute for Oncology, Hematology, and Cell Therapy, Tehran University of Medical Sciences, Tehran, Iran

**Keywords:** ubiquitin-specific peptidases, USPs, cancer, lymphoma, non-Hodgkin, Hodgkin

## Abstract

**Background:**

Ubiquitin-specific peptidases (USPs), also known as deubiquitinating enzymes (DUBs), play a crucial role in maintaining cellular homeostasis by selectively removing ubiquitin molecules from targeted proteins. This process affects protein stability, subcellular localization, and activity, thereby influencing processes such as DNA repair, cell cycle regulation, and apoptosis. Abnormal USP activities have been linked to various diseases, including cancer. Emerging evidence in lymphoma studies highlights the significance of USPs in controlling signaling pathways related to cancer initiation and progression and presents them as potential therapeutic targets.

**Aim:**

This study aimed to elucidate the multifaceted roles of USPs in lymphoma.

**Methods:**

This systematic review was conducted according to the Preferred Reporting Items for Systematic Reviews and Meta-Analyses (PRISMA) guidelines. Articles published in English up to May 2023 were retrieved from PubMed, Web of Science, and Scopus. The inclusion criteria focused on studies investigating the role of USPs in lymphoma cancer, involving human subjects or relevant lymphoma cell lines, exploring molecular mechanisms and signaling pathways, and assessing diagnostic or prognostic value.

**Results:**

After the selection process, 23 studies were selected for analysis. USPs were found to affect various aspects of lymphoma development and progression. Specific USPs were identified with roles in cell-cycle regulation, apoptosis modulation, drug resistance, DNA repair, and influence of key oncogenic pathways, such as B cell receptor (BCR) signaling.

**Conclusion:**

This systematic review underscores the emerging role of USPs in lymphoma and their potential as therapeutic targets. Inhibitors of USPs, such as USP14 inhibitors, show promise in overcoming drug resistance. The dynamic interplay between USPs and lymphoma biology presents an exciting opportunity for future research and the development of more effective treatments for patients with lymphoma. Understanding the intricate functions of USPs in lymphoma offers new insights into potential therapeutic strategies, emphasizing the significance of these enzymes in the context of cancer biology.

## 1 Introduction

Ubiquitin-specific peptidases (USPs), also known as deubiquitins (DUBs), play an important role in maintaining cellular homeostasis by selectively cleaving ubiquitin molecules from targeted proteins. This deubiquitination process affects protein stability, subcellular localization, and activity, allowing complex cellular processes, such as deoxyribonucleic acid (DNA) repair, cell-cycle regulation, resistance to infection, and apoptosis. Given the diverse nature of these enzymes, it is not surprising that their abnormal activities have been linked to many human diseases, including cancer (Bhattacharya et al., 2020; Gatti et al., 2020; Qin et al., 2022; Ramakrishna et al., 2015; Yuan et al., 2018).

In the field of lymphoma, emerging evidence indicates the significant influence of USPs, which play important roles in the cancer process. These enzymes control important signaling pathways involved in the initiation and progression of lymphoma, including the regulation of survival, growth, and immunity. As researchers have uncovered the subtleties of these interactions, USPs have emerged as promising therapeutic targets in lymphoma (Hariri and St-Arnaud, 2021; Rojo-Arreola et al., 2020; Shen et al., 2023).

The ubiquitin–protease system (UPS) is likely involved in human brain development since a defect in the gene encoding the E3 ligase E6-AP has been directly implicated in the cause of Angelman syndrome. The UPS operates through several enzymatic steps, including ubiquitin ligases that bind ubiquitin molecules to target proteins and USPs that degrade the molecules and determine the fate of the tagged proteins (Bachiller et al., 2020; Crawford and Irvine, 2013; Lescouzeres and Bomont, 2020; Morrow et al., 2015; Thapa et al., 2020; Yang et al., 2016).

USPs are part of the UPS and contribute to the balance between protein synthesis and degradation. Disruption of this balance can lead to abnormal or damaged cells, resulting in cancer and other diseases. In lymphoma, disruption of the protein degradation pathway can lead to uncontrolled cell proliferation, apoptosis evasion, and a boost of the immune system (Crawford and Irvine, 2013; Kukharsky et al., 2022; Liu et al., 2023; McKinnon and Tabrizi, 2014).

The repertoire of deubiquitination enzymes is broad and diverse, with more than 100 members having different substrate specificities and cellular localizations. Various enzymes have been implicated in the development of lymphoma. For example, USP9X, a DUB known for its role in DNA repair and cell cycle, is overexpressed in certain lymphoma subtypes and contributes to cell survival and disease resistance. Conversely, USP7, a key regulator of p53 stability, plays a role in promoting lymphomagenesis through its ability to inhibit p53-mediated apoptosis (Li et al., 2021; Li et al., 2022; Park et al., 2022; Pawlak et al., 2021; Qi et al., 2020; Qian et al., 2015; Toloczko et al., 2017; Wang et al., 2019; Zhan et al., 2017; Zhou et al., 2018).

Another member of the USP family, USP15, is indirectly involved in nuclear factor kappa B (NF-κB) signaling. NF-κB is a regulator of inflammation and the immune system and is often dysregulated in lymphoma. USP15 potentiates key components of the NF-κB pathway, leading to activation and subsequent oncogenicity. These examples illustrate the subtle and complex roles of deubiquitination enzymes in the development of lymphoma biology (Zhang et al., 2015; Zhong et al., 2021; Zhou et al., 2020).

Lymphomagenesis is a multifactorial process that is usually driven by the activation of specific signals that promote the growth and survival of cancer cells. USPs have emerged as important regulators because of their ability to regulate the stability and function of proteins involved in these pathways (DeKroon et al., 2018; Jakos et al., 2020; Jiang and Cong, 2016).

One of the most studied pathways in lymphoma is the B-cell receptor (BCR) signaling pathway. Dysfunction of this pathway can lead to uncontrolled B-cell proliferation and lymphoma development. Here, USPs such as OTU deubiquitinase (OTUB1) and CYLD were identified as key regulators that exert their potential by controlling the degradation of key signals in the BCR pathway. For example, OTUB1 supports B-cell survival by stabilizing B-cell lymphoma 6 (BCL6), a transcriptional repressor associated with lymphomagenesis (De Santis et al., 2022; Jiao et al., 2020; Schneider et al., 2016).

The interaction between ubiquitin-specific peptidases and lymphoma provides an interesting explanation for the development of the cancer microenvironment. This review assessed the complex functions of UPS in signaling, protein stability, and immune system regulation during lymphomagenesis.

## 2 Materials and methods

### 2.1 Data sources and search strategies

This study was conducted according to the Preferred Reporting Items for Systematic Reviews and Meta-Analyses (PRISMA) guidelines (Moher et al., 2015). We have included articles published in English until May 2023. Three databases, namely, PubMed, Web of Science, and Scopus, were searched for relevant studies. The exact search strategies used in this study are presented in Table 1. EndNote version 21 was used to manage and deduplicate the retrieved articles and the screening process.

**TABLE 1:** Queries used to systematically search the databases.

Database	Search strategy
PubMed	((Lymphoma Cancer[Title/Abstract] OR lymphatic cancer[Title/Abstract] OR lymphoid neoplasm[Title/Abstract] OR lymphoproliferative disorder[Title/Abstract] OR Hodgkin lymphoma[Title/Abstract] OR non-Hodgkin lymphoma[Title/Abstract] OR b-cell lymphoma[Title/Abstract] OR t-cell lymphoma[Title/Abstract] OR diffuse large b-cell lymphoma[Title/Abstract] OR follicular lymphoma[Title/Abstract] OR mantle cell lymphoma[Title/Abstract] OR Burkitt lymphoma[Title/Abstract] OR primary mediastinal b-cell lymphoma[Title/Abstract] OR anaplastic large cell lymphoma[Title/Abstract] OR cutaneous lymphoma[Title/Abstract] OR extra nodal lymphoma[Title/Abstract] OR lymphomatous infiltration[Title/Abstract] OR lymphadenopathy[Title/Abstract] OR malignant lymphoma[Title/Abstract] OR lymphoma subtypes[Title/Abstract]) OR (Lymphoma Cancer[MeSH Terms])) AND (((((((((((((((Ubiquitin Specific Proteases[Title/Abstract]) OR (Ubiquitin-Specific Peptidase[Title/Abstract])) OR (Peptidase, Ubiquitin-Specific[Title/Abstract])) OR (Ubiquitin Specific Peptidase[Title/Abstract])) OR (Ubiquitin-Specific Peptidases[Title/Abstract])) OR (Peptidases, Ubiquitin-Specific[Title/Abstract])) OR (Ubiquitin Specific Peptidases[Title/Abstract])) OR (Ubiquitin-Specific Protease[Title/Abstract])) OR (Protease, Ubiquitin-Specific[Title/Abstract])) OR (Ubiquitin Specific Protease[Title/Abstract])) OR (Ubiquitin-Specific Protease Family[Title/Abstract])) OR (Protease Family, Ubiquitin-Specific[Title/Abstract])) OR (Ubiquitin Specific Protease Family[Title/Abstract])) OR ((Ubiquitin-Specific Peptidase[MeSH Terms]) OR (Ubiquitin Specific Proteases[MeSH Terms]))))
Scopus	(TITLE-ABS-KEY ( lymphoma AND cancer) OR TITLE-ABS-KEY (lymphatic AND cancer) OR TITLE-ABS-KEY (lymphoid AND neoplasm) OR TITLE-ABS-KEY (lymphoproliferative AND disorder) OR TITLE-ABS-KEY (hodgkin AND lymphoma) OR TITLE-ABS-KEY (non-hodgkin AND lymphoma) OR TITLE-ABS-KEY (b-cell AND lymphoma) OR TITLE-ABS-KEY (t-cell AND lymphoma) OR TITLE-ABS-KEY (diffuse AND large AND b-cell AND lymphoma) OR TITLE-ABS-KEY (follicular AND lymphoma) OR TITLE-ABS-KEY (mantle AND cell AND lymphoma[) OR TITLE-ABS-KEY (burkitt AND lymphoma) OR TITLE-ABS-KEY (primary AND mediastinal AND b-cell AND lymphoma) OR TITLE-ABS-KEY (anaplastic AND large AND cell AND lymphoma) OR TITLE-ABS-KEY (cutaneous AND lymphoma[) OR TITLE-ABS-KEY (extra AND nodal AND lymphoma) OR TITLE-ABS-KEY (lymphomatous AND infiltration) OR TITLE-ABS-KEY (lymphadenopathy) OR TITLE-ABS-KEY (malignant AND lymphoma) OR TITLE-ABS-KEY (lymphoma AND subtypes) ) AND (( TITLE-ABS-KEY ( ubiquitin AND specific AND proteases) OR TITLE-ABS-KEY (ubiquitin-specific AND peptidase) OR TITLE-ABS-KEY (peptidase, AND ubiquitin-specific) OR TITLE-ABS-KEY (ubiquitin AND specific AND peptidase) OR TITLE-ABS-KEY (ubiquitin-specific AND peptidases) OR TITLE-ABS-KEY (peptidases, AND ubiquitin-specific) OR TITLE-ABS-KEY (ubiquitin AND specific AND peptidases) OR TITLE-ABS-KEY (ubiquitin-specific AND protease) OR TITLE-ABS-KEY (protease, AND ubiquitin-specific) OR TITLE-ABS-KEY (ubiquitin AND specific AND protease) OR TITLE-ABS-KEY (ubiquitin-specific AND protease AND family) OR TITLE-ABS-KEY (protease AND family, AND ubiquitin-specific) OR TITLE-ABS-KEY (ubiquitin AND specific AND protease AND family) ))
Web of Science	(Lymphoma Cancer) OR (lymphatic cancer) OR (lymphoid neoplasm) OR (lymphoproliferative disorder) OR (Hodgkin lymphoma) OR (non-Hodgkin lymphoma) OR (b-cell lymphoma) OR (t-cell lymphoma) OR (diffuse large b-cell lymphoma) OR (follicular lymphoma) OR (mantle cell lymphoma) OR (Burkitt lymphoma) OR (primary mediastinal b-cell lymphoma) OR (anaplastic large cell lymphoma) OR (cutaneous lymphoma) OR (extranodal lymphoma) OR (lymphomatous infiltration) OR (lymphadenopathy) OR (malignant lymphoma) OR (lymphoma subtypes) (Topic) AND (Ubiquitin Specific Proteases) OR (Ubiquitin-Specific Peptidase) OR (Peptidase, Ubiquitin-Specific) OR (Ubiquitin Specific Peptidase) OR (Ubiquitin-Specific Peptidases) OR (Peptidases, Ubiquitin-Specific) OR (Ubiquitin Specific Peptidases) OR (Ubiquitin-Specific Protease) OR (Protease, Ubiquitin-Specific) OR (Ubiquitin Specific Protease) OR (Ubiquitin-Specific Protease Family) OR (Protease Family, Ubiquitin-Specific) OR (Ubiquitin Specific Protease Family) (Topic)

### 2.2 Inclusion and exclusion criteria

The following inclusion and exclusion criteria were used to select appropriate articles:

*Inclusion criteria*: 1) studies that investigated the role of USPs in lymphoma cancer; 2) studies published in English; 3) original articles; 4) studies focusing on human subjects or relevant lymphoma cell lines; 5) articles that provide insights into the involvement of USPs in the pathogenesis, progression, diagnosis, prognosis, or treatment of lymphoma; 6) studies that explore the molecular mechanisms or signaling pathways associated with USPs in lymphoma; 7) articles that investigate the potential of USPs as therapeutic targets in lymphoma; 8) studies examining the diagnostic or prognostic value of USPs in lymphoma.

*Exclusion criteria*: 1) studies not directly related to the role of USPs in lymphoma cancer; 2) non-English publications; 3) non-original articles such as review studies, conference abstracts, editorials, or letters; 4) animal or *in vitro* studies not directly applicable to lymphoma; 5) duplicate articles or studies with insufficient data; 6) studies that primarily focus on other types of cancer without substantial inclusion of lymphoma.

## 3 Results

### 3.1 Study selection

**FIGURE 1:**
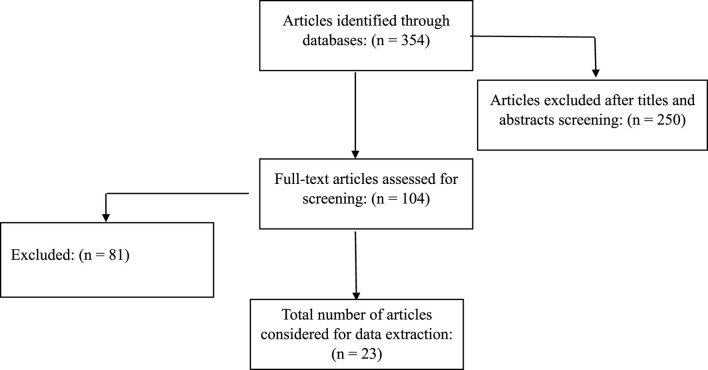
Flowchart of the study selection procedure. The flow diagram was made based on the PRISMA guidelines.

In total, 354 records were extracted from all databases (Figure 1 represents the study selection approach). After excluding duplicates, non-original (including review articles, letters, and editorials), and irrelevant articles, 23 studies were selected for an accurate evaluation of the role of USPs in lymphoma cancer (Table 2).

**TABLE 2:** Summary of the data extracted from the articles included in this study.

No.	Author(year)	Gene symbol	Role, function, and remarks	Cellular location	Target	Inhibitor	References
1	Alzhanova D (2021)	USP7	p53 deubiquitination, regulation of p53 accumulation, localization of p53 to the cytoplasm, its diminished transcriptional activity, and ORF45-USP7 protein–protein interaction.	Cytoplasm	p53	ORF45	Alzhanova et al. (2021)
2	Chappell DL (2023)	USP9X	Potential interactor of vPK (viral protein kinase), regulation of viral reactivation and infectious virion production, and regulation of viral kinase activity.	N/A	vPK	N/A	Chappell et al. (2023)
3	Davis MI (2016)	USP2	Cell cycle, DNA repair, and tumor cell growth. Induction of growth arrest in cells. HR-mediated DNA repair.	N/A	Cyclin D1	ML364	Davis et al. (2016)
4	Delforoush M (2016)	USP14	New targets for proteasome inhibitors in DLBCL.	Cytoplasm	N/A	N/A	Delforoush et al. (2017)
5	Zhao C (2021)	USP18	Key immune gene in EN DLBCL, modulation of the MAPK pathway, activation of dendritic cells (aDCs), modulation of DC-mediated immune responses, development of EN DLBCL, correlation with the top three immune gene sets, type I IFN response, and regulatory T cells (Tregs).	N/A	MAPK	N/A	Zhao et al. (2021)
6	Wu YY (2022)	USP7	Drastic effect on ABC-DLBCL, but not GCB-DLBCL cells. Regulation of BCR-signaling. Stabilization of WDR5 and MLL2.	N/A	BCR signaling (MYC and IRF4)	N/A	Wu et al., 2022b
8	Ruf IK (2009)	UBP43 (USP18)	Regulation of interferon signaling, regulator of IFN-α, and STAT phosphorylation.	N/A	ISG15, STAT1, and IFN-α.	N/A	Ruf et al. (2009)
9	Robinson JE (2020)	USP24	SMZL pathogenesis, diagnosis, and staging of SMZL.	N/A	N/A	N/A	Robinson et al. (2020)
10	Peng W (2020)	USP9X	Regulation of Mcl-1, caspase-3, Bak, and cytochrome C activity. Cell proliferation, apoptosis, progression, and development of DLBCL and its associated pathways.	N/A	Mcl-1 i	N/A	Peng et al. (2020)
11	Fu Y (2021)	USP12	CD4 ^+^ T-cell differentiation, activation, and proliferation, but not CD8 ^+^ T cell. Stabilization of BCL10. Activation of the NF-κB signaling pathway, and stabilization of the TCR complex.	N/A	NF-κB	N/A	Fu et al. (2021)
12	Huang G (2022)	USP9X	Proliferation, cell cycle, cell apoptosis, angiogenesis, cell migration, tumor formation, and tube formation in HUVECs. Mediating cyclin D1 (CCND1)-mediated SOX11 expression. Regulating CDK4, CDK6, PCNA, and P21 protein level.	N/A	SOX11 and CCND1.	N/A	Huang et al., 2022b
13	Hulse M (2021)	USP2a	Mediates fatty acid synthase (FASN) stability. Interaction with FASN.	N/A	FASN protein	ML364	Hulse et al. (2021)
14	Hunter JE (2022)	USP1	Regulation of CHK1 protein levels, mediating resistance to CHK1 inhibition, correlation with USP14, and affecting the clonogenic potential.	N/A	CHK1	ML323	Hunter et al. (2022a)
15	Kamran DES (2023)	USP28, USP36 and USP37	Positive association with the overexpression of c-MYC in the ABC subtype of DLBCL but not with the GCB subtype.	N/A	c-MYC	Peptidyl disruptor	Kamran et al. (2023)
16	Li C (2018)	USP34	Association with older age, GCB subtype, multiple extra-nodal involvement and high IPI scores and DFS of DLBCL.	Nucleus of DLBCL cells; the cytoplasm and/or nucleus of reactive lymphoid hyperplasia cells.	NF-κB signaling	N/A	Li et al. (2018)
17	Li XY (2023)	USP1	Cell growth, cell-cycle arrest, and autophagy. USP1 directly interacted with MAX, a MYC-binding protein, and maintained the stability of MAX, which promoted the transcription of MYC target genes.	Nucleus and cytoplasm.	MAX, a MYC-binding protein.	Pimozide	Li et al. (2023)
18	Lin YH (2019)	Usp44	Regulator of cell cycle, gene expression, and genomic stability. Role in hematopoietic and immune systems. Maintenance of hematopoietic stem cell numbers. Immunoglobulin class switching. Antibody response to immunization.	Nuclear	N/A	N/A	Lin et al. (2019)
19	Ma H (2021)	USP21	Cell proliferation. No obvious effect on cell death. Stabilizing EZH2 (a protein required for germinal center formation and lymphoma formation).		EZH2	siRNA	Ma et al. (2021)
21	Qu CJ (2018)	USP8, USP9X, and USP15	USP8: SMO-mediated regulation of TRAF6. USP9X: cell survival and chemoresistance. USP15: stabilization of MDM2 and regulating p53 function; tumor-cell survival.	N/A	K48-Ub on SMO.	N/A	Qu et al. (2018)
22	Ruan GX (2022)	Usp39	Transition of pre-pro-B to pro-B cells in the bone marrow, B-cell development, regulation of immunoglobulin gene rearrangement in a spliceosome-dependent manner, and progression of B cell lymphomagenesis.	At the interface between U4/U6 and U5 snRNPs (spliceosome component).	c-Myc; immunoglobulin gene rearrangement in a spliceosome-dependent manner and chromatin interaction at the Igh locus.	N/A	Ruan et al. (2022a)
23	Tu R (2021)	USP29	Coordinates with MYC and HIF1α transcriptional programs. Regulator of tumor cell metabolism. Tumorigenesis.	N/A	MYC and HIF1α.	26 S proteasome inhibitor, MG132.	Tu et al. (2021)
24	Wei T (2016)	USP2	Cell apoptosis via p53 signaling. Interaction with Mdm2 protein level and p53 transcriptional activity. Therapeutic resistance.	N/A	Mdm2, p53, and p21.	N/A	Wei et al. (2016)

#### 3.1.1 USPs regulate cell cycle progression by regulating cycle-related factors

USP2 stabilizes cyclin D1, which participates in homologous recombination (HR)-mediated DNA repair. In numerous cell types, it promotes cell-cycle progression from G1 to S. USP2 becomes active or overexpressed in several cancers, such as mantle cell lymphoma, a subtype of non-Hodgkin’s lymphoma. It has been demonstrated that ML364 (small-molecule USP2 and USP8 inhibitor) induces cell-cycle arrest through cyclin D1 degradation by inhibiting USP2.

In CD4^+^ T cells, USP12 stabilizes B-cell lymphoma/leukemia 10 (BCL10), leading to the activation of the NF-κB signaling pathway. USP12 plays an important role in the differentiation, activation, and proliferation of the CD4^+^ T cell phenotype, but it is not observed in CD8^+^ T cells. According to a recent study, USP12 plays a critical role in prostate cancer through deubiquitination of androgen receptors to increase Ak strain transforming (AKT) signaling. Knockdown of USP12 in HeLa cells leads to cell-cycle arrest and reduced transcription of BMI-1, c-Myc, and cyclin D2; therefore, USP12 may adjust cell-cycle progression (Fu et al., 2021).

USP44 is a significant regulator of the cell cycle, DNA repair, and gene expression. USP44 deficiency in the Emu-Myc mouse B-cell lymphoma model causes early lethality (Lin et al., 2019).

#### 3.1.2 The role of USPs in apoptosis

Several key factors have been recognized to play important roles in apoptosis, one of the most important being the tumor suppressor gene p53, and its activity inhibits the generation of tumors. USP2 is described as an oncogene due to its capability to inhibit apoptosis. USP2a has been investigated in numerous cancers, and it has been revealed that higher expression of USP2a is related to advanced stages. In advanced cutaneous T-cell lymphoma (CTCL), the expression of USP2a was lower than that of normal T-lymphocytes, suggesting that it is a tumor suppressor in CTCL, unlike in solid cancers. Wei et al. demonstrated that the knockdown of USP2 causes increased apoptosis after PUVA treatment in MyLa 2000, suggesting that USP2 can display therapeutic resistance. They showed that p53 activation can promote USP2 induction. They also illustrated that USP2 stabilized mouse double minute 2 homolog (Mdm2), but more evidence is needed to link USP2 deubiquitinase activity with Mdm2-p53 signaling. They also explained that USP2 may play an anti-apoptotic role in CTCL and exert a reverse effect in PUVA (Wei et al., 2016).

Kaposi’s sarcoma-associated herpesvirus (KSHV) expresses a series of open reading frames (ORFs) to prevent premature apoptosis and support its viability. Considering that USP7 is a p53 deubiquitinase, KSHV ORF45 inhibits the interactions between USP7 and p53 by binding to p53. As a result, the accumulation and localization of p53 decrease in the cytoplasm, and its transcriptional function decreases.

Under normal circumstances, USP7 binds to human double minute 2 (HDM2) via the death domain-associated protein death-associated protein 6 (DAXX), which prevents the self-ubiquitination of HDM2 and causes the degradation of p53 by HDM2 accumulation. However, in the context of DNA damage, HDM2 dissociates from the USP7–DAXX complex, resulting in self-ubiquitination and subsequent degradation of HDM2, and then USP7 binds to p53 (Alzhanova et al., 2021). USP15 stabilizes MDM2 and regulates p53 function, thus boosting tumor cell survival (Qu et al., 2018).

Based on a recent experiment, USP9X overexpression was found associated with diffuse large B-cell lymphoma (DLBCL) development and progression. USP9X expression was four times greater in malignant cells, including Farage and Pfeiffer cells, than in normal B cells. Inhibition of this USP using siRNA downregulates the expression of Mcl-1 and upregulates the expression of caspase-3, Bak, and cytochrome C; therefore, knockdown of USP9X induces cell apoptosis and decreases cell proliferation in DLBCL (Peng et al., 2020).

Furthermore, USP9X upregulation plays a key role in the formation of mantle cell lymphoma (MCL) via different pathways, including enhanced cell proliferation and cell cycle, inhibition of cell death, and induction of angiogenesis. USP9X is present at high levels in both peripheral blood mononuclear cells (PBMCs) and MCL cells of patients with MCL. Because USP9X affects cell migration and angiogenesis by elevating CCND1-mediated SOX11 expression, USP9X suppression leads to the downregulation of USP9X protein levels in Z-138 and Jeko-1 cell lines and inhibits tumor development in mice *in vivo*. In addition, USP9X knockdown shortens the duration of the S stage; reduces the expression of cyclin-dependent kinase 4 (CDK4), cyclin-dependent kinase 6 (CDK6), and proliferating cell nuclear antigen (PCNA); and increases P21 protein expression (Huang et al., 2022a).

USP21 was shown to be overexpressed in the DLBCL lymphoid tissue compared with matching healthy tissues and cell lines such as A20 and SU-DHL-4. It increases the growth of DLBCL cells through cysteine 221 (the catalytic site of USP21), but it does not affect cell death. In addition, USP21 participates in the development of lymphoma through the stabilization of enhancer of zeste homolog 2 (EZH2), which is required for generating germinal centers and lymphoma tumors. There is a positive correlation between the elevated expression of USP21 and high mortality in patients; therefore, small interfering RNA (siRNA) knockdown of USP21 and consequent reduction of tumor cell proliferation may be a promising treatment for DLBCL (Ma et al., 2021).

LIM homeobox 2 (LHX2) is an oncogene that promotes malignancy in breast cancer and pancreatic ductal adenocarcinoma. LHX2 adjusts USP18 expression in cancers with poor prognosis. In addition, the reduction of the expression of the immune gene USP18 can decrease the number of activated dendritic cells (aDC), resulting in poor prognosis (Zhao et al., 2021). In addition, a disorder in USP18 expression in Burkitt lymphoma can lead to interferon (IFN)-stimulated gene expression. Therefore, it is likely that USP18 regulates IFN-I-associated immune responses to develop extranodal diffuse large B-cell lymphoma (EN DLBCL) with poor prognosis. The mitogen-activated protein kinase (MAPK) pathway is a possible downstream pathway of USP18 that plays a role in cellular processes such as proliferation, differentiation, and apoptosis. Overall, USP18 plays a potential role via the MAPK pathway and aDCs in EN DLBCL. In addition to USP12, it regulates the MAPK signaling pathway because its knockout leads to impaired MAPK activity in cells (Zhao et al., 2021).

USP7 regulates various activities, including DNA repair, cell cycle progression, protein localization, and apoptosis. USP7 is overexpressed in lymphomas in dogs, and P5091 is an inhibitor that has cytotoxic effects in canine lymphoma and cancer cells. USP7i likely stabilizes the p53 protein and, therefore, may cause apoptosis (Pawlak et al., 2021).

#### 3.1.3 USP function in the DNA repair pathway

USP44 plays an important role in DNA repair through its DUB catalytic function on the histones H2B and H2A and promotes gene silencing. USP44 deficiency and overexpression are prone to errors in chromosome separation, aneuploidy, and cancer; for example, its overexpression was noted in T-cell acute lymphoblastic leukemia (Lin et al., 2019). USP34 expression increases in the germinal center B-cell-like (GCB) subtype of DLBCL cases, and USP34 is correlated with Wnt signaling activation by destabilization of β-catenin. In addition, USP34 responds to DNA damage and is a downstream target of mutant ataxia telangiectasia in DNA damage (Li et al., 2018).

UBP43, also known as USP18, is encoded by an interferon-stimulated gene (ISG) and functions as a remover of the ISG15 polypeptide from ISGylated proteins. This protease negatively regulates IFN-I signaling by blocking STAT phosphorylation, leading to a reduction in the induction of ISGs. This inhibition occurs via the direct displacement of Janus kinase 1 (JAK1) from the IFN-α/β receptor. A reduction in the IFN-inducible expression of UBP43 leads to a notable delay in the negative feedback regulation of type-I interferon signaling in Burkitt lymphoma (BL) cell lines (including Akata A.2 and A.15), regardless of their Epstein–Barr virus (EBV) status (Ruf et al., 2009).

#### 3.1.4 USPs and drug resistance

The use of drugs that inhibit proteasome (mainly bortezomib) activity in multiple myeloma is currently a standard therapy. One way of targeting the proteasome is through USPs such as USP14 for different cancer types, including lymphoma, and even cancer cells resistant to bortezomib (Delforoush et al., 2017).

USP24 has anti-apoptotic tumoral activity in myeloma cells and is overexpressed in drug-resistant cells. It was also found that enhanced USP24 expression was regularly observed in the interfollicular zones of splenic marginal zone lymphoma (SMZL) patients. Given its role in regulating tumor microenvironment signaling, USP24 has been identified as a potential therapeutic target (Robinson et al., 2020).

USP1 can act as an oncogene and is highly expressed in DLBCL patients. High expression of USP1 is associated with poor prognosis in DLBCL. USP1 plays an important role in the rituximab/chemotherapy resistance of DLBCL via deubiquitination of MAX (highly expressed in DLBCL cells). Knockdown of USP1 leads to inhibition of cell proliferation, cell-cycle arrest, reduced MAX/MYC, and autophagy in DLBCL cells (Li et al., 2023).

Some members of the USP family are considered possible targets of anticancer agents, such as USP7, which targets c-Myc. USP7 inhibition destabilizes the CHK1 protein, resulting in AML cells being sensitized to the chemotherapeutic factor cytarabine. Targeting USP1 may also be another therapeutic strategy for MYC-related tumors. Eµ-Myc/cRel^−/−^ lymphomas have downregulated the expression of USP1 and checkpoint kinase 1 (CHK1) protein that results in resistance to treatment by specific CHK1i. The loss of the CHK1 pathway is related to the downregulation of CHK1, and USP1 inhibition with ML323 can contribute to CHK1 inhibitor resistance (Hunter et al., 2022b).

USP39 elimination in the B-cell lineage prevented the transition from pre-pro-B cells to pro-B cells in the bone marrow, resulting in substantial reduction in mature B cells in peripheral tissues. USP39 ablation in Em-Myc mice successfully controls the expansion of malignant pre-B cells, lowered B-cell lymphomagenesis, and boosted survival. Because the spliceosome component of USP39 has a role in B-cell development and in controlling the rearrangement of immunoglobulin genes, targeting USP39 could represent a promising therapeutic strategy for treating B-cell lymphoma (Ruan et al., 2022).

USP37 expression is positively associated with c-MYC expression in activated B-cell-like-DLBCL (ABC-DLBCL) but not with its GCB subtype. USP37 stabilizes c-MYC and causes unusual cell growth. USP22 and USP36 regulate the cellular turnover of c-MYC in breast cancer, and c-MYC expression is stabilized by USP28 and USP37 in colon carcinoma and lung cancer, respectively. Other USPs, including USP13, USP16, USP17, and USP22, can maintain c-MYC expression in the GCB subtype of DLBCL (Kamran et al., 2023).

Viruses such as Kaposi’s sarcoma-associated herpesvirus (KSHV) control deubiquitination. KSHV is the causative agent of Kaposi’s sarcoma, primal effusion lymphoma, and the plasmablastic shape of multicentric Castleman disease. Here, USP9X plays a pro-viral role, and its depletion prevents virus reactivation and the production of infectious virions (Chappell et al., 2023).

#### 3.1.5 USPs as targets for drug development in cancer prevention

USP29 stabilizes MYC and hypoxia-inducible factor 1-alpha (HIF1α); therefore, it enables tumor cells to respond to both normoxia and hypoxia. Knockout of Usp29 significantly prolongs the survival of tumor-bearing mice by reducing the expressions of MYC and HIF1α in neuroblastoma and B-cell lymphoma. Therefore, USP29 can be used as a new therapeutic target for treating cancer (Tu et al., 2021).

USP7 expression was significantly increased in three cancer types, including DLBCL. ABC-DLBCL is a subtype of non-Hodgkin’s lymphoma with a poor prognosis; its survival is related to the activation of BCR signaling. USP7 stabilizes WD repeat-containing protein 5 (WDR5) and mixed-lineage leukemia 2 (MLL2) (parts of lysine-specific methyltransferase complex) in ABC-DLBCL cells. The expression of USP7 is upregulated in ABC-DLBCL cells and downregulated in GCB-DLBCL cells; therefore, USP7 inhibition has a serious effect on ABC-DLBCL cells, contrary to that observed in GCB-DLBCL cells. ABC-DLBCL cells upregulate the expression of some components of BCR signaling, and USP7 inhibition decreases the expression of upregulated parts of BCR signaling and plays an important role in therapeutic targets (Wu et al., 2022a).

Latent membrane protein 1 (LMP1) is expressed in EBV-associated lymphomas. USP2a is detected in LMP1-positive Burkitt’s lymphoma (BL) cells and mediates the stabilization of fatty acid synthase (FASN). FASN expression is notably correlated with distant lymph node metastasis. USP2a inhibition through ML364 decreased FASN levels and inhibited the proliferation of LMP1-positive BL cell lines. Targeting USP2a is an effective strategy for future investigation of ML364 treatment in malignancies with LMP1-positive expression (Hulse et al., 2021).

The fact that we only considered three databases (PubMed, Web of Science, and Scopus) may prove to be a limitation to our study. In addition, the exclusion of studies published in other languages may constitute another limitation of our study.

## 4 Conclusion

In conclusion, this systematic review delved into the emerging and intricate role of USPs in lymphoma, shedding light on their potential as therapeutic targets. The landscape of research on USPs in lymphoma has revealed a novel and promising avenue for understanding and potentially treating this complex group of hematologic malignancies. Through an extensive literature review, this article highlights the diverse functions of USPs, which span critical cellular processes such as cell-cycle regulation, apoptosis, DNA repair, and drug resistance. A comprehensive understanding of how USPs influence these processes in the context of lymphoma is essential for researchers and clinicians seeking to unravel the intricacies of this disease.

As our understanding of the intricate roles of USPs in lymphoma deepens, the prospect of developing more effective and targeted treatment options for patients with lymphoma becomes increasingly feasible. The dynamic interplay between USPs and lymphoma biology presents an exciting opportunity for further research and clinical exploration to improve the outcomes and quality of life for patients affected by this heterogeneous group of malignancies. Future investigations in this field may unveil novel and innovative strategies for combating lymphoma, reaffirming the significance of USPs as a focal point of interest in cancer research and therapy.

## 5 Key points

1. USPs and cellular homeostasis: USPs, also known as deubiquitinase (DUBs), are essential for maintaining cellular balance by selectively removing ubiquitin molecules from target proteins. The deubiquitination process influences protein stability, subcellular localization, and activity, contributing to critical cellular processes such as DNA repair, cell-cycle regulation, immunity, and apoptosis.
2. USPs and human diseases: dysregulation of USP activities is associated with various human diseases, including cancer. Abnormalities in these enzymes can lead to disruption of cellular processes, resulting in conditions such as cancer. The versatile nature of USPs makes them a potential therapeutic target.
3. USPs in lymphoma: in lymphoma, USPs play a significant role in controlling the signaling pathways that affect cancer initiation and progression. They regulate critical factors involved in cell survival, growth, and immunity. The abnormal activities of USPs are linked to the development of lymphoma, highlighting their potential as therapeutic targets.
4. Ubiquitin–proteasome system (UPS): understanding the effects of USPs on lymphoma requires a better understanding of the ubiquitin–proteasome system (UPS). The UPS regulates protein degradation and function through a series of enzymatic steps, including ubiquitin ligases and USPs. Aberrations in this system can lead to cancer and other diseases by disrupting the balance between protein synthesis and degradation.
5. Diverse roles of USPs: with more than 100 members, USPs exhibit diverse substrate specificity and cellular localizations. Specific USPs are implicated in the development of lymphoma, influencing processes such as DNA repair, cell-cycle progression, apoptosis, and immune response regulation.
6. Targeting USPs for therapy: given the intricate roles of USPs in lymphomagenesis, they are potential targets for therapeutic intervention. Inhibiting specific USPs could disrupt cancer-promoting pathways, thereby leading to new treatment strategies for lymphoma and related malignancies.

## Data Availability

The original contributions presented in the study are included in the article/Supplementary Material; further inquiries can be directed to the corresponding authors.

## Nomenclature

USPs: ubiquitin-specific peptidases.
DUBs: deubiquitinases.
UPS: ubiquitin–proteasome system.
DNA: deoxyribonucleic acid.
DLBCL: diffuse large B-cell lymphoma.
NF-κB: nuclear factor Kappa B.
BCR: B-cell receptor.
CTCL: cutaneous T-cell lymphoma.
IFN: interferon.
EBV: Epstein–Barr virus.
LMP1: latent membrane protein 1.
GCB: germinal center B-cell-like.
ABC: activated B-cell-like.
HDM2: human double minute 2.
DAXX: death-associated protein 6.
CDK4: cyclin-dependent kinase 4.
CDK6: cyclin-dependent kinase 6.
PCNA: proliferating cell nuclear antigen.
EZH2: enhancer of zeste homolog 2.
siRNA: small interfering RNA.
MAPK: mitogen-activated protein kinase.
IFN: interferon.
JAK1: Janus kinase 1.
CHK1: checkpoint kinase 1.
HIF1α: hypoxia-inducible factor 1-alpha.
MLL2: mixed-lineage leukemia 2.
WDR5: WD repeat-containing protein 5.
OTUB1: OTU deubiquitinase.
HR: homologous recombination.
AKT: Ak strain transforming.
Mdm2: mouse double minute 2 homolog.
HDM2: human double minute 2.
